# Ginsenoside compound K induces ferroptosis via the FOXO pathway in liver cancer cells

**DOI:** 10.1186/s12906-024-04471-9

**Published:** 2024-04-25

**Authors:** Jiaxin Chen, Zhuoshi Wang, Jinghao Fu, Yuesong Cai, Haoyi Cheng, Xinmu Cui, Manqing Sun, Mingyue Liu, Xuewu Zhang

**Affiliations:** College of Medicine, Yanbian University, Yanji, China

**Keywords:** Ginsenoside CK, Ferroptosis, Liver cancer, FOXO pathway, Anticancer

## Abstract

Liver cancer is a common malignant tumor worldwide, traditional Chinese medicine is one of the treatment measures for liver cancer because of its good anti-tumor effects and fewer toxic side effects. Ginsenoside CK (CK) is an active component of ginseng. This study explored the mechanism by which CK induced ferroptosis in liver cancer cells. We found that CK inhibited the proliferation of HepG2 and SK-Hep-1 cells, induced ferroptosis of cells. Ferrostatin-1, an ferroptosis inhibitor, was used to verify the role of CK in inducing ferroptosis of liver cancer cells. Network pharmacological analysis identified the FOXO pathway as a potential mechanism of CK, and western blot showed that CK inhibited p-FOXO1. In cells treated with the FOXO1 inhibitor AS1842856, further verify the involvement of the FOXO pathway in regulating CK-induced ferroptosis in HepG2 and SK-Hep-1 cells. A HepG2 cell–transplanted tumor model was established in nude mice, and CK inhibited the growth of transplanted tumors in nude mice, p-FOXO1 was decreased in tumor tissues, and SLC7A11 and GPX4 expressions were also down-regulated after CK treatment. These findings suggested that CK induces ferroptosis in liver cancer cells by inhibiting FOXO1 phosphorylation and activating the FOXO signaling pathway, thus playing an antitumor role.

**Supplementary Information:**

The online version contains supplementary material available at 10.1186/s12906-024-04471-9.

## Introduction

Liver cancer is a common tumor of the digestive system and a serious threat to human health. Hepatocellular carcinoma (HCC) is the main histological type of liver cancer and accounts for approximately 90% of liver cancer cases [1]. Liver cancer is characterized by concealment of onset rapid progression, late diagnosis, and frequent recurrence. Chemotherapy, immunotherapy, biological targeting and traditional Chinese medicine are common systematic treatments for liver cancer [2]. However, due to the strongly toxic and side effects, and with the increasing of drug concentration and treated time, the drug resistance was enhanced and seriously degraded the patient’s quality of life, so it is urgent to seek an anti-liver cancer drug with less toxicity and side effects. At present, traditional Chinese medicine and its active ingredients has the advantages of less toxic side effects and many targets, it has attracted much attention of researchers in recent years. Ginseng is a rare Chinese herbal medicine, which has a powerful effect [3]. Ginsenoside CK (CK) is an effective component of ginseng, which has anti-cancer, anti-diabetes, anti-inflammation, anti-allergy, anti-angiogenesis, anti-aging, liver protection and other effects [4, 5]. Our research group previously found that CK regulates Bclaf1 under hypoxia conditions to affect the HIF-1α-mediated glycolysis pathway, thus inhibiting the growth of liver cancer cells through in vitro and in vivo experiments [6]. CK Induced endoplasmic reticulum stress and apoptosis in human liver cancer cells by regulating STAT3 [7]. CK induced mitochondrial apoptosis in human liver cancer cells through Bclaf1-mediated modulation of ERK signaling [8]. These results indicated that CK had anti-liver cancer outcome. However, the molecular mechanism of CK against liver cancer needed to be further improved. Many studies currently have shown that traditional Chinese medicine achieves anticancer effects by inducing ferroptosis of tumor cells. For example, Dihydroartemisinin caused ferroptosis in tumor cells by disrupting the intracellular redox balance [9], Baicalin induced ferroptosis in osteosarcomas through a novel Nrf2/xCT/GPX4 regulatory axis [10], Piperlongumine rapidly induced the death of human pancreatic cancer cells mainly through the induction of ferroptosis [11]. However, the mechanism of CK as the active ingredient of Chinese medicine ginseng, on ferroptosis of liver cancer cells hasn’t clear.

Ferroptosis is an iron-dependent cell death method discovered by Stockwell et al. in 2012, caused by excessive lipid peroxidation and subsequent plasma membrane rupture [12]. Ferroptosis can be induced by either exogenous or endogenous pathways. The exogenous pathway is initiated by inhibiting cysteine/glutamate transporters or activating ferric transporters. The endogenous pathway is activated by blocking intracellular antioxidant enzymes such as glutathione peroxidase 4 (GPX4). The main point of ferroptosis is that the fenton reaction occurs after Fe^2+^ overload in cells, which causes excessive consumption of GPX4, and lipid peroxidation of cell membranes, affecting the flow, permeability, and finally leading to cells rupture and death. It is a regulatory cell death process [13]. GPX4 and SLC7A11 are the core regulatory proteins in ferroptosis. GPX4 degrades some small molecule peroxides and lipid peroxides in the cell, such as catalyzing GSH to produce GSSG, thereby inhibiting lipid peroxidation [14]. SLC7A11 is a multichannel transmembrane protein that mediates intracellular transport of cysteine, a precursor molecule of glutathione. GPX4 and SLC7A11 play an important role in maintaining intracellular oxidative stress and inhibiting the occurrence of ferroptosis. System xc- promotes the glutamate and cystine exchange across the plasma membrane. SLC7A11 is mainly responsible for transport activity and can specifically recognize and bind glutamic acid and cystine to complete transport [15]. When the system xc-transport activity is affected, the activity of SLC7A11 receives inhibition, GSH levels can be reduced, thereby affecting GPX4 function and induce cells ferroptosis [16]. The SLC7A11-GSH-GPX4 axis is considered the main cellular system that defends against ferroptosis. Targeting the cell death process is a common approach in cancer therapy, and ferroptosis is also considered as a novel target for anticancer therapy.

Therefore, in this study, we conducted the experiments in vitro and in vivo and network pharmacology analysis to explore the role and mechanism of CK in inducing liver cancer ferroptosis. These findings will provide a theoretical and experimental basis for the development and use of CK as an anti-liver cancer drug.

## Materials and methods

### Experimental cells

HepG2 (No. BNCC338070) and SK-Hep-1 (No. BNCC341844) were purchased from BeNa Culture CollectionKey (Beijing, China). QSG-7701 (No. CL0264) was purchased from Fenghui Biotechnology Co., LTD. (Changsha, Hunan, China).

#### Experimental drugs

CK was purchased from Shanghai Yuanye Biotechnology Co., LTD. (No. B21045), HPLC ≥ 98%, CAS number: 39262-14-1. 5-Fluorouracil (5-Fu) was purchased from Shanghai Yuanye Biotechnology Co., LTD. (No. B25419).

#### Experimental animals

The female BALB/c nude mouse aged 4–5 weeks and weighing 18–22 g (No.: 2,013,001,829,418) was purchased from Shanghai Lingchang Co., LTD. Experimental animal production License: SCXK (Shanghai) 2013-0018.

#### Reagents

Dulbeccos modified Eagle’s medium (DMEM), Roswell Park Memorial Institute (RPMI), Penicillin-Streptomycin, Fetal Bovine Serum were purchased from Gibco (Grand Island, NY, USA); Cell Counting Kit-8 (CCK-8) was purchased from APE×BIO (Houston, USA); ECL chemiluminescence kit was purchased from Millipore (Billerica, Massachusetts, USA); GSH content detection kit and MDA content detection kit were purchased from Solarbio (Beijing, China); Antibodies p-FOXO1, FOXO1, GPX4 were purchased from Abcam (Cambridge, MA, USA); β-actin was purchased from Bioss (Beijing, China); SLC7A11 was purchased from Thermo (Shanghai, China); Horseradase labeled goat anti-rabbit IgG and horseradase labeled goat anti-Mouse IgG were purchased from Zhongshan Jinqiao Co., LTD. (Beijing, China). Ferrostatin-1 and AS 1,842,856 were purchased from MCE (Shanghai, China); C11 BODIPY 581/591 lipid peroxidation fluorescent probe was purchased from Maokang organism (Shanghai, China); Fe^2+^ probe was purchased from Tongren Chemistry (Shanghai, China).

#### Cell proliferation assay

HepG2 and SK-Hep-1 cells were cultured with culture medium (DMEM: fetal bovine serum: penicillin-streptomycin = 100:1:1) in a humidity incubator (37 ℃, 5% CO_2_). QSG-7701 cells were cultured with culture medium (RPMI: fetal bovine serum: penicillin-streptomycin = 100:1:1) in a humidity incubator (37 ℃, 5% CO_2_). When the cells reached the logarithmic growth phase, CCK-8 was used to detect cell proliferation ability, and 100 µL cell suspension was added into the wells of the 96-well plate, so that the cell density in each hole was 5 × 10^3^ cells/mL. After the cells were attached to the wall, the cells were divided into the following groups: control group, CK (20 µM, 40 µM, 60 µM), positive control group 5-Fu (10 µM), each group was set up with 3 compound wells, each group was added with 10 µL CCK-8 solution, and incubated for 1 h. OD values of each well solution were detected at 490 nm wavelength by enzyme-labeled instrument (Bio-Tek; San Jose, CA, USA), and the data were recorded to calculate the cell growth rate.

Cell growth rate (%) = (group experimental group OD value - blank OD value) / (negative control group OD value - blank OD value) × 100%.

#### FerroOrange intracellular Fe^2+^ fluorescence detection

FerroOrange working fluid was configured to avoid light. HepG2 and SK-Hep-1 with logarithmic growth stage were taken, and the cells were washed with serum-free culture medium for 3 times, according to the groups: CK (20 µM, 40 µM,60 µM) and 5-Fu (10 µM) groups were administered and then cultured in a 5% CO_2_ incubator at 37 ℃. After 48 h, the medium was discarded and washed with HBSS and serum-free medium for 3 times. 5 mM of FerroOrange working liquid was added to the petri dish and cultured in the incubator. Photographs were taken 30 min later under a fluorescence microscope (400×) (Olympus; Japan).

#### Detection of liperfluo lipid peroxide content

The lipid peroxidation fluorescent probe C11 BODIPY 581/591 was diluted to 50 mM in serum-free medium, with 1 mL of C11 BODIPY 581/591 working solution added to each well, and incubated at 37℃ for 1 h without light. Then, the cells were washed three times with serum-free medium to completely remove C11 BODIPY 581/591 that did not enter the cells. The cells were photographed by fluorescence microscope (400×), and the fluorescence intensity was analyzed by Image J software.

#### MDA detection

1 × 10^6^ cells/mL HepG2 and SK-Hep-1 were placed into EP tubes and the supernatant was centrifuged. After 500 µL extract was added into the test tube, the cells were broken repeatedly by ultrasound for 30 times, then divided into EP tubes, centrifuged (8 000 g, 10 min, 4 ℃), the supernatant was collected and placed on ice, and the enzymic spectrometer was preheated for more than 30 min in advance. Distilled water was added to the 96-well plate and zeroed. In the experimental group, 300 µL of MDA working liquid, 100 µL of distilled water, and 100 µL of MDA kit reagent 3 were added to each tube. In the control group, 300 µL MDA working liquid and 100 µL MDA kit reagent 3 were added. Cover the mixture tightly with a water bath at 100 ℃ for 120 min, immediately place it on ice for cooling, centrifuge (10 000 g, 10 min), absorb an appropriate amount of supernatant and place it in the 96-well plate, and test the absorbance values at 450 nm, 532 nm and 600 nm of each hole with an enzyme labeling instrument. The content of MDA in the sample to be measured was calculated by the formula in the product description.

#### GSH detection

After 48 h of CK in the experimental group (20 µM, 40 µM, 60 µM), 5-Fu group (10 µM) and vehicle group, cells were collected and the cell concentration was adjusted to 1 × 10^6^ cells/mL. After the collected cells were cleaned twice with PBS, a reagent was added to the suspended cells, and the cells were placed in liquid nitrogen at -80℃ and water bath at 37 ℃ for repeated freezing and thawing for 3 times. The supernatant (8 000 g, 10 min) was centrifuged by high-speed centrifuge, reagent 2 was placed in a 37 ℃ water bath for 35 min. Blank hole (adding 20 µL distilled water, 140 µL reagent 2, 40 µL reagent 3), sample hole (adding 20 µL sample, 140 µL reagent 2, 40 µL reagent 3), standard curve hole (adding 20 µL standard, 140 µL reagent 2, 40 µL reagent 3), 3 repeat holes were set up in each group, and after mixing, the absorbance was measured at 412 nm by normal temperature. The absorbance was recorded as A determination and A blank. Make standard curves. The standard product (1 mg/mL) was diluted by reagent 1 into a concentration gradient of 300 µg/mL, 200 µg/mL, 100 µg/mL, 50 µg/mL and 25 µg/mL, and the standard curve was prepared. After mixing each well, it was left for 2 min at room temperature. The absorbance was measured at 412 nm using A preheated enzymograph, denoted as A standard. ΔA = A determination - A blank, ΔA standard = A standard -A blank. According to the specification, add ΔA to the standard curve formula to calculate the sample concentration, and then calculate the GSH content based on the number of cells.

#### Establishment of transplanted tumor model in nude mice

HepG2 subcutaneous transplantation tumor model in nude mice was established, and corresponding drug intervention was performed in groups when the tumor volume of nude mice reached 100 mm^3^, and the inhibitory effect of drugs on tumor in nude mice was observed. The safety of the compound in vivo was tested according to the designed dose, with 3 animals in each group, administered for 1 week and observed for 1 week. The concentration of HepG2 cells was adjusted to 1 × 10^7^ cells/mL, and the 0.1 ml cell suspension was inoculated into 32 nude mice respectively, tumor volume was measured with a vernier caliper, and the grouped drug intervention began when the tumor volume reached 100 mm^3^. One intervention every 1 day; during the process, the weight and tumor data were measured every three days until the tumor volume reached the sampling was terminated at 1 500 mm^3^. The group design was as follows: vehicle control group (normal saline group) ginsenoside CK (5 mg/kg, 10 mg/kg, 20 mg/kg). All nude mice were killed after anesthesia, and tumor chunks were taken to measure their length and width, and the volume and growth inhibition rate were calculated. The anesthetic was 20% urethane, 200 µL/20 g intraperitoneally injected. By inhibiting the activity of acetylcholinesterase, urethane caused the accumulation of acetylcholine, which affected the normal nerve conduction of experimental animals and anesthetised them.

Graft volume of tumor (TV) (cm^3^) = length × width^2^ × 1/2.

Tumor growth inhibition ratio (%)= (average tumor weight in normal group - average tumor weight in drug administration group) / average tumor weight in normal group × 100%.

#### Immunohistochemistry

Tissue wax blocks were made by transplanting tumor in nude mice. After the sections were soaked in xylene for 10 min, dehydrated in gradient, the antigen repair solution was added, and the sections were kept in a slightly boiling state for 30 min. 2 drops of 3% hydrogen peroxidation-methanol solution were added to the sections for 10 min, and then 200 µL 5% BSA was added to the sections for 20 min. Each slice was added with 100 µL primary antibody GPX4 (1: 200), SLC7A11 (1:200), incubated at 37 ℃ in a wet box for 2 h, then dropped with 80 µL enhancer and placed for 30 min, added with 100 µL of secondary antibody, placed in an incubator at 37 ℃ for 30 min, add 50 µL DAB solution, applied the hemoxylin dye solution to the tissue sections for 10 min, soaked in distilled water, dehydrated with ethanol gradient, transparented with xylene for 20 min, then added neutral gum to seal the tablets.

#### Western blot

HepG2 and SK-Hep-1 cells at logarithmic growth stage were treated with drugs to extract cell or tumor tissue proteins. The protein concentration was determined by BCA method, and the loading was calculated according to the sample concentration. Sodium dodecyl sulfate polyacrylamide gel electrophoresis (SDS-PAGE) (100 V, 120 min), SDS-PAGE gels were trimmed according to the molecular weight of the target protein and then cropping the appropriate sizes of polyvinylidene fluoride (PVDF) membranes and transferring them (100 V, 30–90 min), then closed the membrane with 5% skim milk, the primary antibody was diluted with TBST: GPX4 (1: 1 000), SLC7A11 (1: 1 000), β-actin (1: 30 000), FOXO1 (1: 2 000), p-FOXO1 (1: 2 000) were incubated overnight at 4 ℃. On the second day, anti-rabbit secondary antibody (1:5 000) was incubated at room temperature for 2 h and ECL luminescent solution was added. Target protein was detected by Bioanalytical Imaging system (Cycloud, Beijing, China).

#### Network pharmacological analysis

Ginsenoside CAS numbers were searched through the organic small molecule bioactivity database (https://pubchem.ncbi.nlm.nih.gov/). The 2D structure sdf format and SMILES encoding of ginsenoside compounds were obtained. Then compounds SDF file was imported SwissTargetPrediction target prediction database (http://www.swisstargetprediction.ch/) and CK SMILES coding was import, the search conditions were limited to human sources to obtain potential drug targets. Then searched keyword “HCC” in GeneCards (https://www.genecards.org/), setted the score is greater than the standard median filter tidy HCC disease targets. In the Omim (https://www.omim.org/) database, filteried Gene symbols dominated by *. Integrated the target points of the two databases and imported the obtained data into Excel tables for standardized processing. The Uniprot database was used to calibrate and integrate drug targets to remove duplicates. FerrDb (http://www.zhounan.org/ferrdb/current/) is a database of ferroptosis, the related genes regulating ferroptpsis were obtained by using this database and exported to Excel table for summary. Through the Venn Diagrams (http://www.bioinformatics.com.cn/static/others/jvenn/example.html) to get the intersection targets of CK treated HCC by regulating iron metabolism. The protein interaction network was constructed through the STRING 11.0 (https://string-db.org/cgi/input.pl). The common targets of CK-HCC-ferroptpsis was enriched by GO function and KEGG signaling pathway enrichment analysis by using R language. (* P < 0.05)

#### Statistical analysis

Data analysis was performed using GraphPad Prism 9.5.1, data are expressed as mean ± standard deviation (SD). And differences between groups were analyzed by one-way analysis of variance and Student’s *t*-test. *P* < 0.05 was considered to be statistically significant.

## Results

### CK inhibited the proliferation of HepG2 and SK-Hep-1 cells

**Fig. 1:**
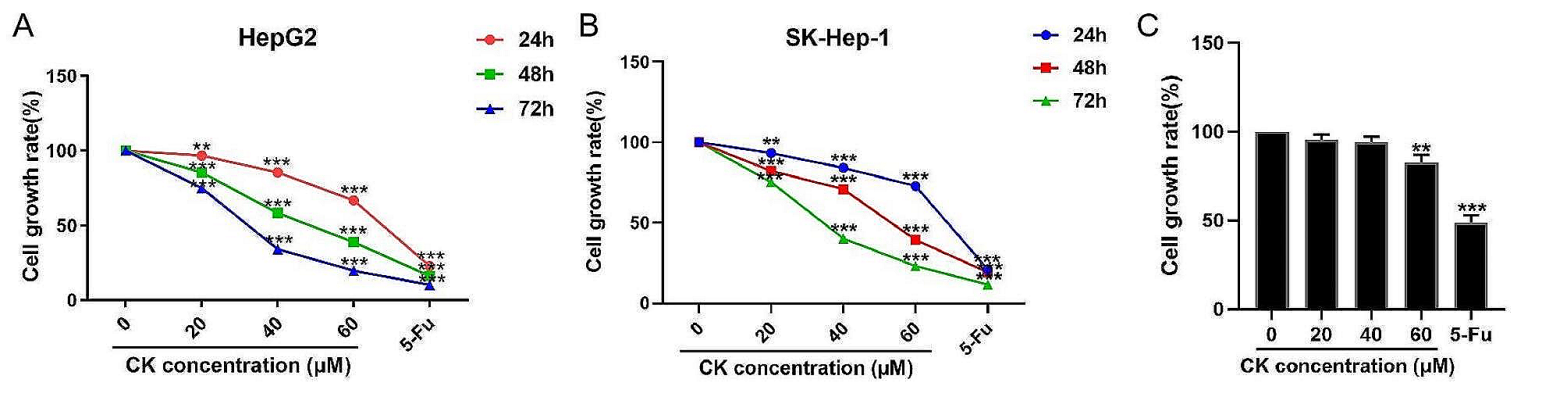
CK inhibited the proliferation of HepG2 and SK-Hep-1 cells. **(A)** Cell growth rates of HepG2 cells treated with CK (20 µM, 40 µM, 60 µM) and 5FU (10 µM) for 24 h, 48 h, and 72 h were detected by CCK-8 assay.** (B) **Cell growth rates of SK-Hep-1 cells treated with CK (20 µM, 40 µM, 60 µM) and 5-Fu (10 µM) for 24 h, 48 h, and 72 h were measured by CCK-8 assay. **(C)** Cell growth rates of QSG-7701 cells treated with CK (20 µM, 40 µM, 60 µM) and 5-Fu (10 µM) for 48 h, were measured by CCK-8 assay. ^**^P < 0.01, ^***^P < 0.001, compared with the vehicle group. Differences between groups were analyzed by one-way ANOVA, and pairwise comparison between groups was performed by *t*-test

HepG2 and SK-Hep-1 cells were treated with CK (20 µM, 40 µM, 60 µM) for 24 h, 48 h and 72 h and the cell growth rate was detected by CCK-8 assay. The growth rates of both cell lines decreased with the increased of CK concentration in a time-dependent manner (*P* < 0.01) (Fig. 1A, B). These results indicated that CK inhibited the proliferation of HepG2 and SK-Hep-1 cell lines in a time-and dose-dependent manner. The IC50 values of CK in HepG2 and SK-Hep-1 cells treated for 48 h were 47.661 µM and 53.141 µM, respectively. Therefore, we selected a concentration of 40 µM and treatment time of 48 h for subsequent experiments. Compared with control group, CK (20 µM, 40 µM) had no significant effect on normal hepatocytes QSG-7701 for 48 h, and cell viability decreased after CK (60 µM) treatment (*P* < 0.01). When the cells were treated with 5-Fu, the viability of the cells decreased significantly (*P* < 0.001) (Fig. 1C). The results indicatied that lower concentrations of CK weren’t toxic to hepatocytes and 5-Fu was more toxic to hepatocytes.

### CK induced ferroptosis of HepG2 and SK-Hep-1 cells

**Fig. 2:**
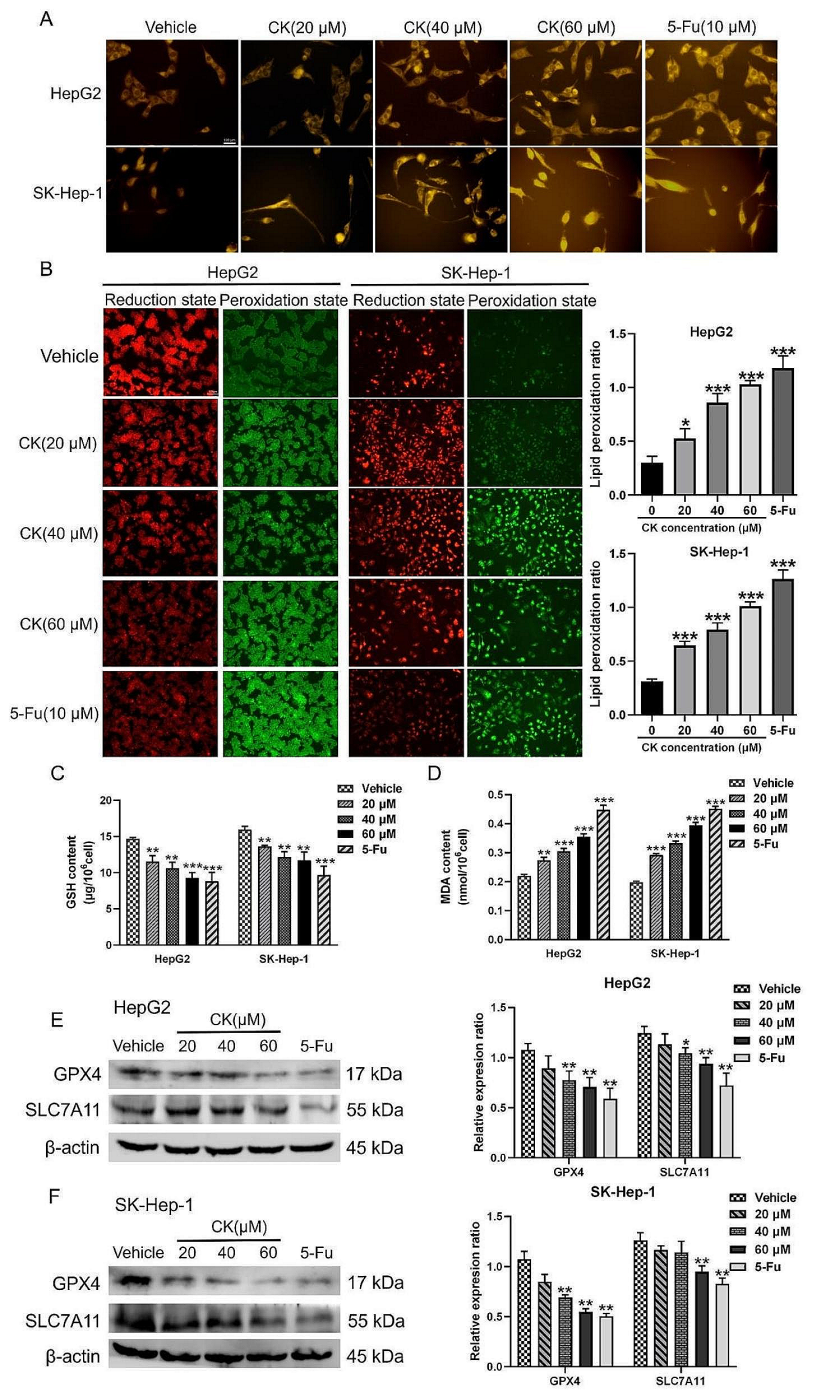
CK induced ferroptosis of HCC cells HepG2 and SK-Hep-1. **(A)** FerroOrange probe was used to detect Fe^2+^ levels in HepG2 and SK-Hep-1 cells (400×). **(B)** The intracellular lipid peroxidation rate of HepG2 and SK-Hep-1 cells (400×). **(C, D)** The GSH and MDA contents in HepG2 and SK-Hep-1 cells were detected. **(E, F)** Expression of ferroptosis-related proteins GPX4 and SLC7A11. ^*^P < 0.05, ^**^P < 0.01, ^***^P < 0.001, compared with the vehicle group. Pairwise comparison between groups was performed by *t*-test

Fe^2+^ has a high oxidation capacity, and it is easy to produce a large number of reactive oxygen species in the Fenton reaction with H_2_O_2_, thus promoting lipid peroxidation [17]. A significant increase in Fe^2+^ content is a typical biochemical feature of cells ferroptosis. FerroOrange probe detection showed that the intracellular Fe^2+^ content in CK (20, 40, 60 µM) and 5-Fu (10 µM) groups was increased compared with the control group (Fig. 2A). Excessive lipid peroxidation is one of the important characteristics of ferroptosis, particularly when phospholipids on the cell membrane are over-oxidized; the integrity of the cell membrane will be damaged, resulting in cell membrane rupture, mediating the occurrence of ferroptosis. A lipid peroxidation probe was used to determine the levels of reductive and oxidizing lipids and the intracellular lipid peroxidation rate was determined. The results showed that the lipid peroxidation rate of cells was significantly increased after CK treatment compared with the control treatment (*P* < 0.001) (Fig. 2B). GSH is the most important antioxidant in the cellular antioxidant system. The intracellular GSH content was significantly decreased after CK treatment compared with the controls (*P* < 0.001) (Fig. 2C). Additionally, MDA levels in cells increased significantly after CK treatment compared with controls (*P* < 0.001) (Fig. 2D). Western blot showed that the expressions of GPX4 and SLC7A11 in the CK treatment group were decreased in a concentration-dependent manner compared with controls (Fig. 2E and F).

### Effect of ferrostatin-1 and CK on the ferroptosis pathway in HepG2 and SK-Hep-1 cells

**Fig. 3:**
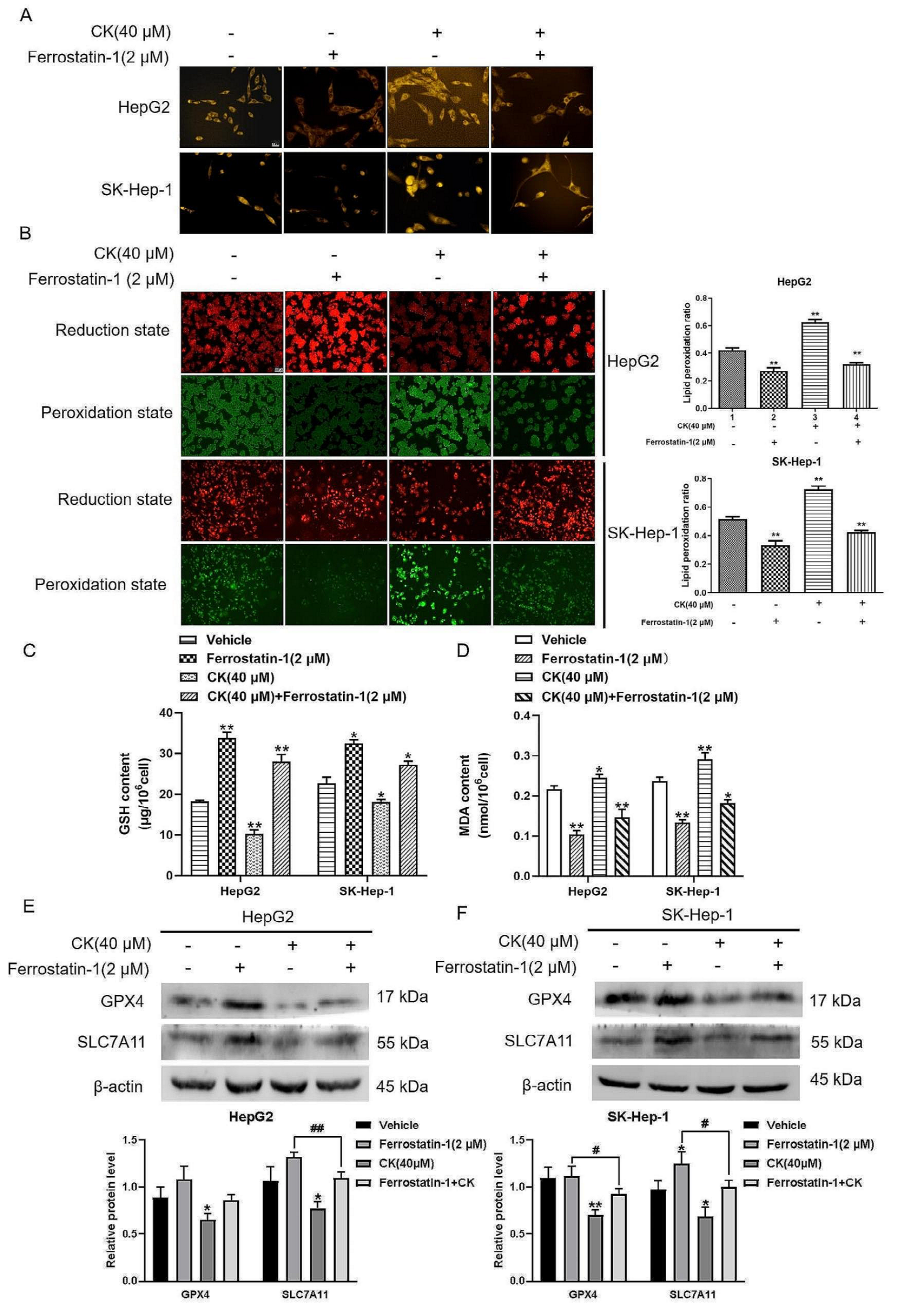
Effect of ferrostatin-1 and CK on the ferroptosis pathway in HepG2 and SK-Hep-1 cells. **(A)** The FerroOrange probe was used to detect Fe^2+^ levels in HepG2 and SK-Hep-1 cells (400×). **(B)** Liperfluo cell lipid peroxide probes were used to examine cells treated with CK (40 µM) and ferrostatin-1 (2 µM). The lipid peroxidation rate of HepG2 and SK-Hep-1 cells was observed by fluorescence microscopy after 48 h (400×). **(C, D)** Effects of ferrostatin-1 (2 µM) and CK (40 µM) on MDA and GSH contents, compared with vehicle group, ^*^P < 0.05, ^**^P < 0.01. **(E, F)** The expression of GPX4 and SLC7A11 after CK and ferrostatin-1 treatment in cells. ^*^P < 0.05, ^**^P < 0.01, compared with the vehicle group; ^#^*P* < 0.05, ^##^*P* < 0.01, the ferrostatin-1 group was compared with the CK + ferrostatin-1 group. Pairwise comparison between groups was performed by *t*-test

To further verify the effect of CK on inducing ferroptosis, we used ferrostatin-1, an inhibitor of ferroptosis. While the level of Fe^2+^ in cells in CK treatment group was up-regulated compared with levels in controls, ferrostatin-1 reduced the level of Fe^2+^ in cells, there was no significant change in Fe^2+^ levels compared with control groups after CK and ferrostatin-1 treatment together. (Fig. 3A). While CK up-regulated the level of intracellular lipid peroxidation, ferrostatin 1 reduced the level of intracellular lipid peroxidation (*P* < 0.01). When CK and ferrostatin 1 were used to treat HepG2 and SK-Hep-1 cells at the same time, the level of intracellular lipid peroxidation did not change significantly compared with levels in the blank control group (Fig. 3B). Compared with the vehicle group, MDA level decreased and GSH level increased in the ferrostatin-1 group, compared with the ferrostatin-1 group, GSH decreased and MDA increased in the CK + ferrostatin-1 group. (Fig. 3C.D). GPX4 and SLC7A11 levels increased after ferrostatin-1 treatment. Compared with the ferrostatin-1 group, the expression level of GPX4 and SLC7A11 was downregulated in CK + ferrostatin-1 group (Fig. 3E and F).

### Network pharmacological analysis indicated CK may induce ferroptosis by affecting the FOXO signaling pathway

**Fig. 4:**
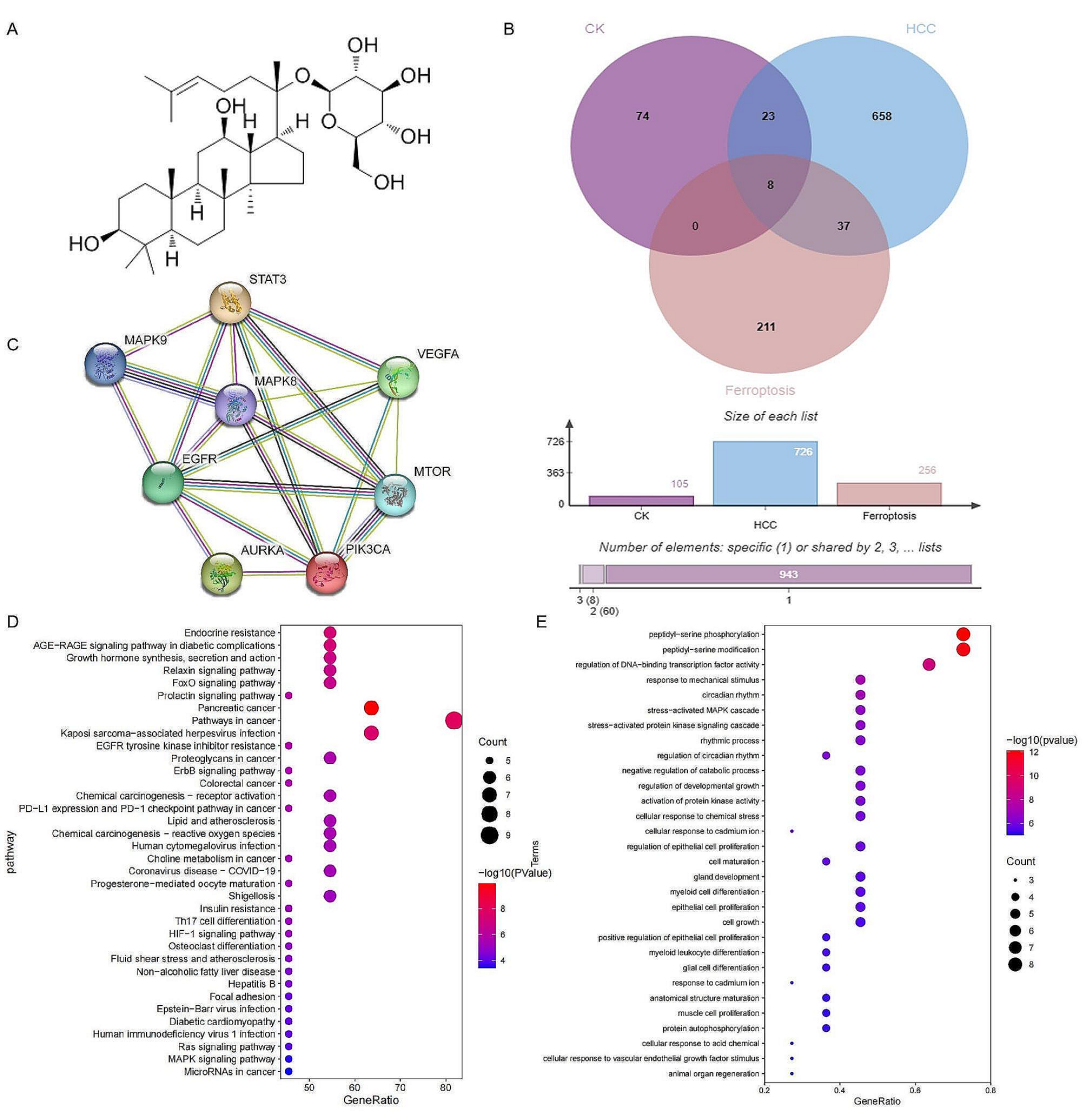
CK might induce ferroptosis by affecting the FOXO signaling pathway. **(A)** The sdf format of the 2D structure of CK compound components; **(B)** Venn map of the intersecting target genes of CK, HCC, and ferroptosis; **(C)** PPI network diagram of common targets of CK regulation of ferroptosis against HCC. **(D)** Bubble map for KEGG pathway enrichment analysis; a common target of CK-HCC-ferroptosis. **(E)**. Bubble map of GO functional enrichment analysis of CK-HCC-ferroptosis common targets

The intersection targets (Fig. 4B) of CK (Fig. 4A) for HCC by ferroptosis were obtained by network pharmacological analysis. Through protein interaction network construction, PIK3CA, EGFR, MTOR, VEGFA, STAT3, AURKA, MAPK8 and MAPK9 were 8 common targets of CK regulating iron death against HCC (Fig. 4C). Thirty-six pathways were identified by KEGG pathway enrichment analysis (Fig. 4D). Among them, we focused on FOXO signaling pathway, FOXO signaling pathway depends on its phosphorylation level of FOXO protein, and GO function enrichment analysis was also significantly enriched for phosphorylation modification of amino acids (Fig. 4E). FOXO proteins are a group of key transcription regulatory proteins involved in a variety of cell biological functions (cell proliferation, apoptosis, metabolism, and aging), and the family members include FOXO1, FOXO3, FOXO4 and FOXO6. The FOXO1 transcription factor is involved in various biological processes, including regulating the cell cycle, promoting apoptosis, and resisting oxidative stress damage. Combining the results of GO functional enrichment analysis and KEGG pathway enrichment analysis, we selected FOXO1 for further exploration.

### CK induced ferroptosis by regulating the FOXO signaling pathway in HepG2 and SK-Hep-1 cells

**Fig. 5:**
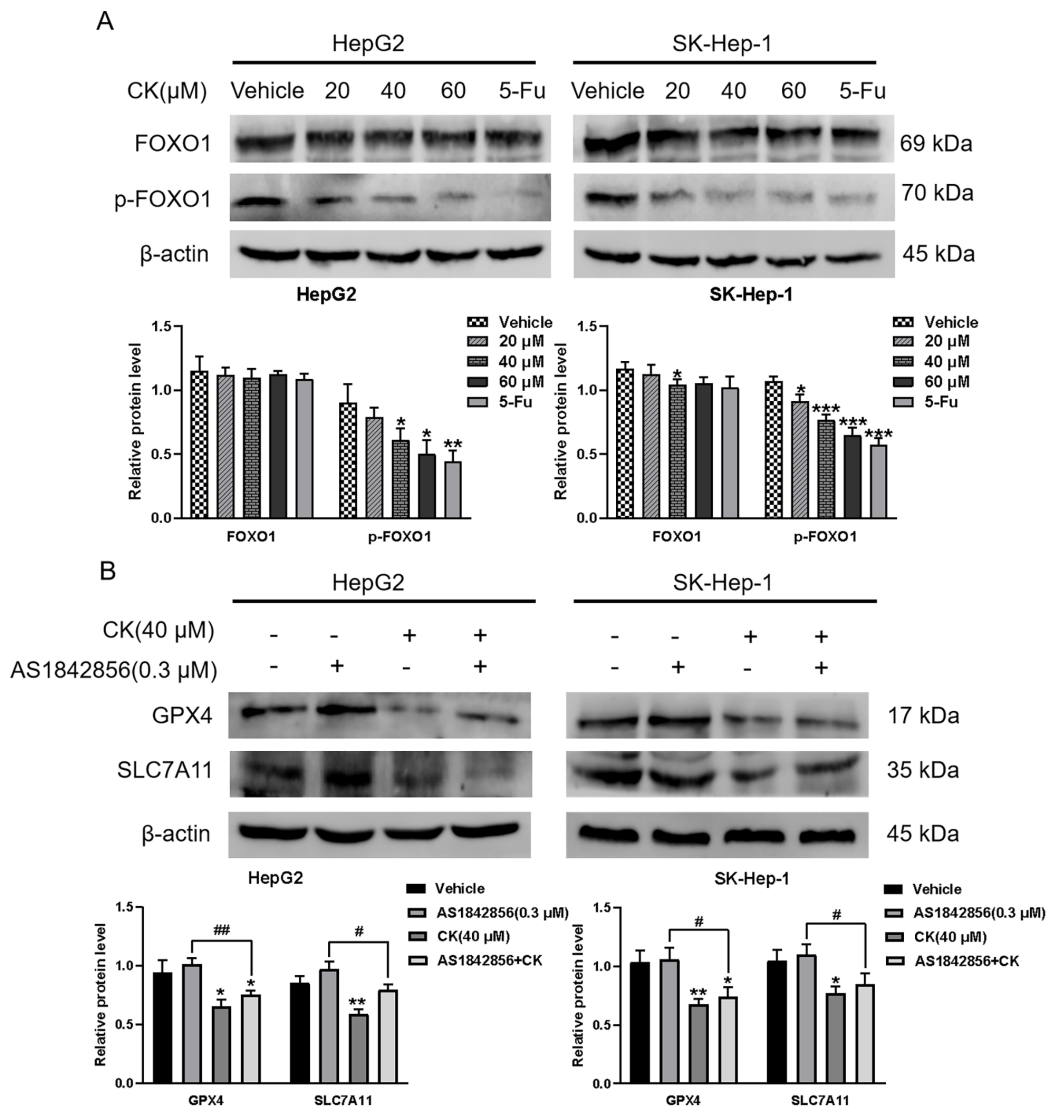
CK induced ferroptosis of HepG2 and SK-Hep-1 cells by regulating the FOXO signaling pathway. **(A) **Western blot was used to detect FOXO pathway-related proteins in cells after 48 h treatment with CK (20 µM, 40 µM, 60 µM) and 5-Fu (10 µM). **(B)** Western blot of ferroptosis-related proteins GPX4 and SLC7A11 in HepG2 and SK-Hep-1 cells treated with AS1842856 (0.3 µM) and CK (40 µM). ^*^P < 0.05, ^**^P < 0.01, ^***^P < 0.001, compared with the vehicle group; ^#^*P* < 0.05, ^##^*P* < 0.01, compared with the CK + AS1842856 group and ferrostatin-1 group. Pairwise comparison between groups was performed by *t*-test

Phosphorylation of FOXO1 is an important mechanism to regulate its activity. When FOXO1 is phosphorylated, FOXO1 is transported from the nucleus to the cytoplasm, thus blocking its transcriptional activity. Unphosphorylated FOXO1 is recruited in large quantities in the nucleus and exerts transcriptional activity [18]. Western blot showed that CK down-regulated p-FOXO1 levels compared with the controls, while the total protein expression level of FOXO1 did not change significantly (*P* < 0.01) (Fig. 5A). These results indicated that CK inhibited FOXO1 phosphorylation in cells and activated the FOXO signaling pathway. To further explore the mechanism of CK inducing ferroptosis in HepG2 and SK-Hep-1 cells, we used the FOXO1 inhibitor AS1842856. CK significantly reduced the expression levels of GPX4 and SLC7A11 in cells and AS1842856 alone had no significant effect on GPX4 and SLC7A11 expression levels. However, when AS1842856 was combined with CK (40 µM), the expressions of GPX4 and SLC7A11 were upregulated compared with levels in the CK group (Fig. 5B). These findings suggested that CK may induce ferroptosis in liver cancer cells through the FOXO pathway.

### CK inhibited the growth of tumors in nude mice derived from HepG2 cells

**Fig. 6:**
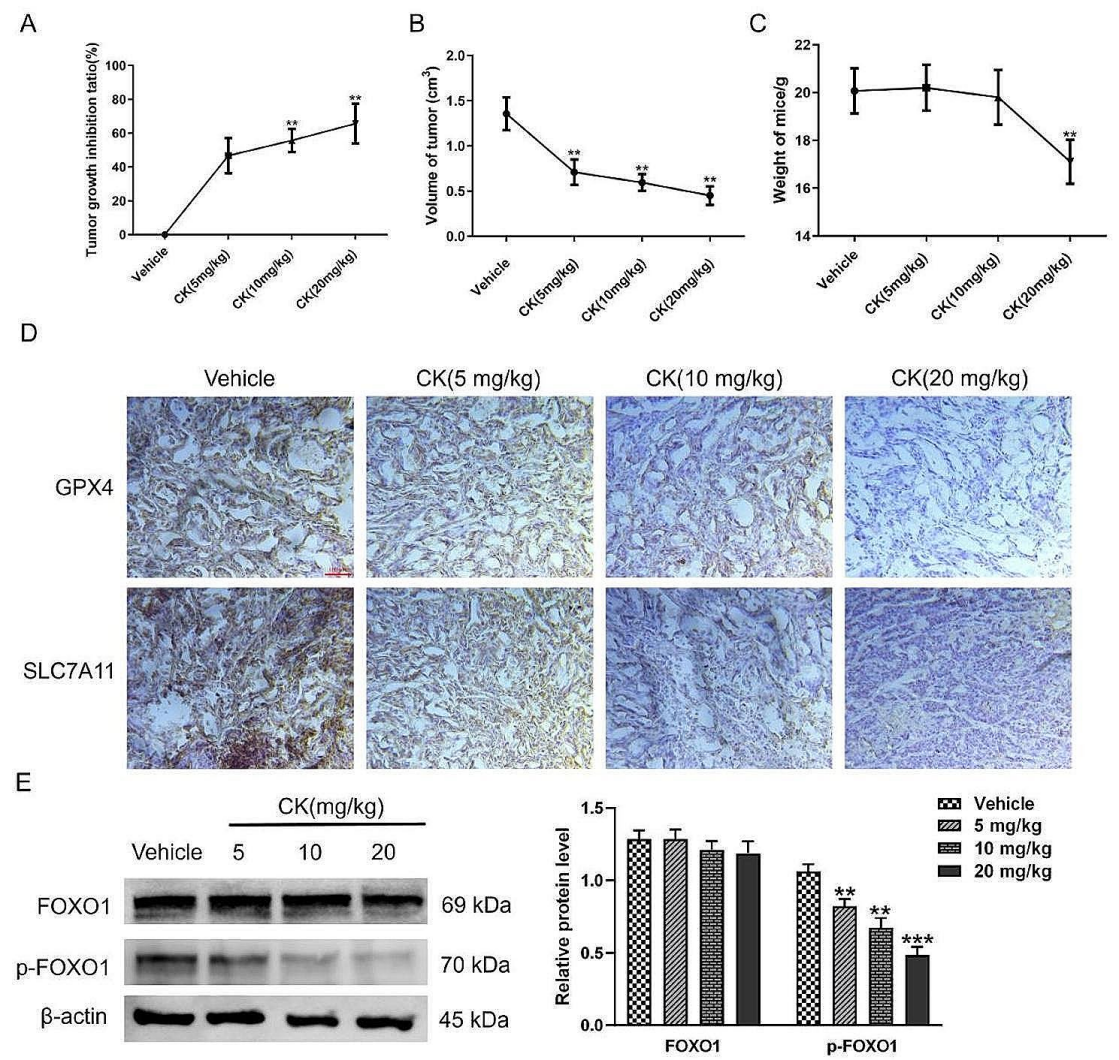
CK inhibited the growth of tumors in nude mice, and CK regulated ferroptosis in tumor tissues by p-FOXO1. **(A) **The tumor growth inhibition rate (%) of nude mice in the indicated groups; **(B)** The tumor volume (cm^3^) of nude mice; **(C)** The weight of tumors (g) in nude mice; **(D)** Immunohistochemical staining was used to detect the effects of CK (5, 10, 20 mg/kg) on GPX4 and SLC7A11 protein expression in tumors derived from nude mice inoculated with HepG2 cells (400×); **(E)** Western blot analysis of tumors from the indicated groups. ^**^P < 0.01, ^***^P < 0.001, compared with the vehicle group. Pairwise comparison between groups was performed by *t*-test

To further explore the anti-tumor effect of CK in vivo, we constructed a nude mouse tumor model inoculated with HepG2 cells and treated mice with CK or vehicle. The tumor volume and tumor weight of nude mice in the CK administration group were decreased compared with the controls, and the tumor inhibition effect of CK increased with the increase of concentration (*P <* 0.01), indicating a concentration-dependent effect (Fig. 6A-C). The expressions of GPX4 and SLC7A11 in tumors were down-regulated by CK compared with vehicle treatment (Fig. 6D). The expression level of phosphorylated FOXO1 also decreased significantly in a concentration-dependent manner (*P* < 0.01), while the levels of FOXO1 remained unchanged (Fig. 6E).

## **Discussion**

Primary liver cancer can be divided into HCC, intrahepatic cholangiocarcinoma, and hepatocellular cholangiocarcinoma on the basis of pathological classification. Among these types, HCC is the most common (85 − 90%) [19–21]. The traditional clinical treatment of HCC was mainly surgery, supplemented by chemoradiotherapy. In recent years, immunotherapy and targeted therapy as novel treatment methods have been widely used in the treatment of HCC. Studies showed that about 1/3 of HCC patients were treated and developed significant drug-resistant within 6 months [22]. Therefore, it is critical to develop new therapeutic methods and drugs to increase the clinical treatment options of HCC patients, and overcome the drug resistance of tumors, thus improve the prognosis of HCC patients. Traditional Chinese medicine is used for the adjuvant treatment of liver cancer has the characteristics of high efficiency and low toxicity. CK is the main active component of Chinese medicine ginseng, which has pharmacological activities and anti-liver cancer, liver protection and inhibition of fatty liver effects [23]. Combining the domestic and foreign research literature and the preliminary researches of our research group, the mechanism of CK on ferroptosis of liver cancer hasn’t clear. In this study, we through the experiments in vitro and in vivo investigated the effect of CK on ferroptosis in HCC cells and investigated the mechanism of CK in inducing ferroptosis in cells through the FOXO signaling pathway.

First CCK-8 assays showed that CK inhibited the proliferation of HepG2 and SK-Hep-1 in a time-and dose-dependent manner. Ferroptosis is a mode of cell death driven by iron ion–dependent excessive lipid oxidation and cell membrane damage. A increasing number of studies suggest that ferroptosis plays a key role in tumor suppression, thus becoming a novel target for tumor therapy. In this study, we treated HepG2 and SK-Hep-1 cells with CK and examined ferroptosis-related indicators. Our research found that CK up-regulated the level of Fe^2+^ in cells, the level of intracellular lipid peroxidation decreased after CK treatment compared with the control treatment. GSH is the main dynamic index reflecting the redox status of cells, the occurrence of ferroptosis depends on lipid peroxidation driven by Fe^2+^, which is also accompanied by a weakening of the antioxidant system, especially the GSH system [24]. As the final product of phospholipid peroxidation of cell membrane, MDA content in the cell can reflect the level of lipid peroxidation to a certain extent. MDA increased significantly and GSH decreased after CK treatment compared with the control treatment. Western blot analysis showed that CK down-regulated the expression of GPX4 and SLC7A11, the key proteins of ferroptosis. To further validated the effect of CK on inducing ferroptosis, we added the ferroptosis inhibitor ferrostatin-1 to detect the indicators associated with ferroptosis. The experimental results showed that ferrostatin-1 inhibited ferroptosis in liver cancer cells, and compared with Ferrostatin-1 group, ferroptosis was more obvious in CK + Ferrostatin-1 group. These findings indicatied that after CK action on liver cancer cells, gathered intracellular Fe^2+^ and triggered the accumulation of lipid peroxide through fenton reaction, and the systemic xc-’s function was affected, the activity of SLC7A11 was inhibited, thus inhibiting GSH synthesis, resulting in reduced GPX4 activity and increased MDA production, which leaded to cells ferroptosis.

To further explore the specific target of CK-induced ferroptosis, we screened out the FOXO signaling pathway by network pharmacology analyse. FOXO1, as an important member of the FOXO transcription factor family [25]. Increasing evidence has shown that FOXO1 can also play a role as a tumor suppressor; it inhibits the proliferation, migration, and invasion of tumor cells, and promotes the apoptosis of tumor cells [26]. Phosphorylation modification of the FOXO1 protein is an important way for the regulation of its protein activity. When FOXO1 is modified by phosphorylation, it promotes the transport of FOXO1 from the nucleus to the cytoplasm, thus losing its transcriptional activity. Conversely, unphosphorylated FOXO1 will be greatly recruited to exert its transcriptional activity in the nucleus. Piao et al. Found that though mutating the phosphorylation sites of FOXO1 protein, inhibiting FOXO1 phosphorylation and increasing the nuclear expression of FOXO1, it can cause G2/M cell cycle arrest in glioblastoma cells, then promote apoptosis and exert anti-tumor effect [27]. It shows that inhibiting the phosphorylation level of FOXO1 is the main way that FOXO1 exerts its anticancer effect. In our study, western blot showed that CK resulted in significantly decreased p-FOXO1 levels. To further explore the effect of CK inducing ferroptosis in liver cancer cells through FOXO pathway, we used AS1842856, a specific inhibitor of FOXO1 protein. AS1842856 directly binds to activated (unphosphorylated) FOXO1 to prevent it from binding to DNA and exert transcriptional activity. Western blot results showed that the expression levels of GPX4 and SLC7A11 in the CK + AS1842856 group were increased compared with the CK group, indicating that CK induced ferroptosis in liver cancer cells through FOXO signaling pathway.

To further verify the mechanism of CK inducing ferroptosis in liver cancer cells, a nude mouse transplantation tumor model of HepG2 was constructed. The results showed that CK inhibited tumor growth. Tumor tissues of the CK treatment group showed inhibited GPX4 and SLC7A11 expression and down-regulated p-FOXO1. The above results indicated that CK inhibition of liver tumor growth in nude mice was related to the inhibition of FOXO1 phosphorylation and the induction of ferroptosis in tumor cells. Additional studies have reported that PI3K/AKT, signaling pathway can promote the phosphorylation of FOXO1, making its transport from nucleus to cytoplasm, losing transcriptional activity [28]. Zhao et al. found that curcumin as an active component of Chinese medicine, reduced FOXO1 phosphorylation by inhibiting the PI3K/Akt signaling pathway, thus maintaining FOXO1 transcriptional activity to inhibit pancreatic cancer cell proliferation [29]. And CK is closely related to PI3K/AKT pathway, and many studies indicated that CK can exert anticancer effects by inhibiting PI3K/AKT signaling pathway to inhibit tumor cells growth and promote tumor cell apoptosis [30, 31]. Therefore, we further speculated that in HCC cells, CK may inhibits the phosphorylation of FOXO1 by inhibiting the PI3K/AKT signaling pathway, and then induces the occurrence of ferroptosis to plays an anticancer effect. Our research group will carry out further research on this problem in the future.

In summary, CK inhibited liver cancer cells proliferation by regulating p-FOXO1 level and induced ferroptosis. This study provided theoretical and experimental basis for the development and application of new therapy based on ferroptosis and CK.

## Conclusion

The research indicated that CK activated the FOXO signaling pathway by inhibiting the p-FOXO1 to induce the occurrence of ferroptosis in liver cancer cells and then exerted the anticancer effect. These findings provided some experimental data and theoretical basis for the antitumor effect of CK.

### Electronic supplementary material

Below is the link to the electronic supplementary material.

Supplementary Material 1

## Data Availability

The all datasets used and analyzed during the current study are available from the corresponding author on reasonable request. Data in this paper were available using publicly available databases: Pubchem(https://pubchem.ncbi.nlm.nih.gov/). SwissTargetPrediction (http://www.swisstargetprediction.ch/). GeneCards (https://www.genecards.org/). Omim (https://www.omim.org/). FerrDb (http://www.zhounan.org/ferrdb/current/).
