# Supplementary material for: Ginsenoside compound K induces ferroptosis via the FOXO pathway in liver cancer cells
Source: BMC Complement Med Ther. 2024 Apr 25;24:174. doi: 10.1186/s12906-024-04471-9 (PMC11044296; doi:10.1186/s12906-024-04471-9)

Each experiment was repeated three times.

Fig.2(HepG2)

GPX4(1)

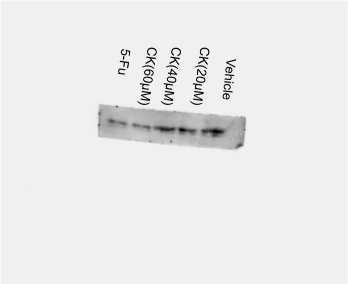

GPX4(2)

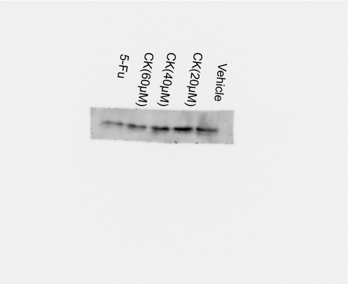

GPX4(3)

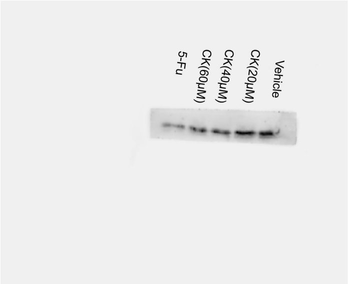

SLC7A11(1)

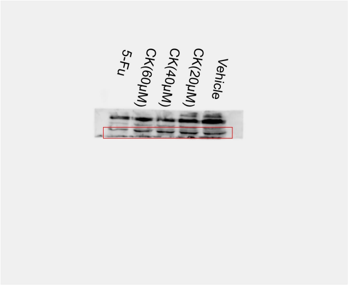

SLC7A11(2)

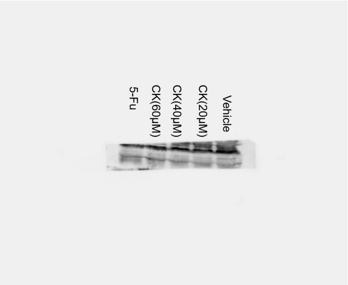

SLC7A11(3)

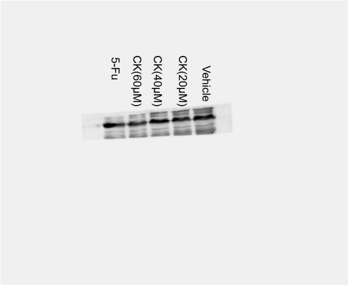

β-actin(1)

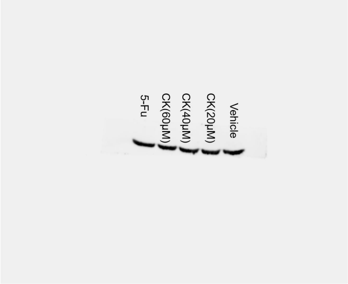

β-actin(2)

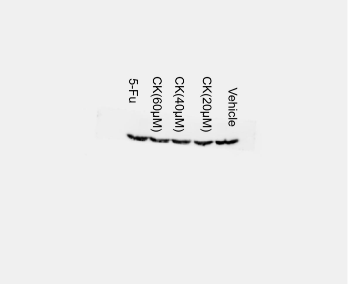

β-actin(3)

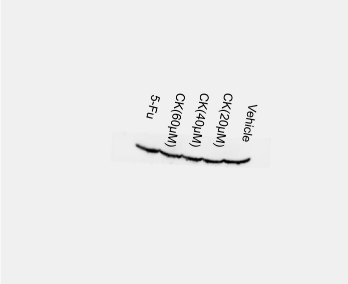

Fig.2(SK-Hep1)

GPX4(1)

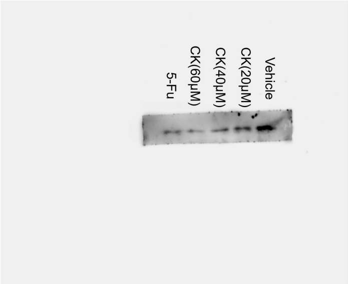

GPX4(2)

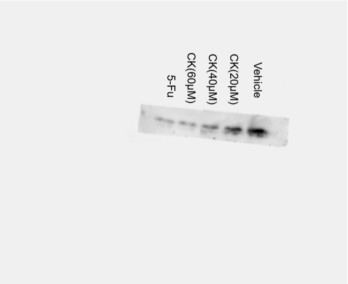

GPX4(3)

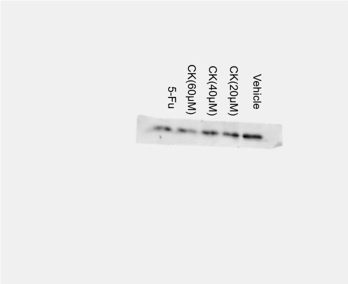

SLC7A11(1)

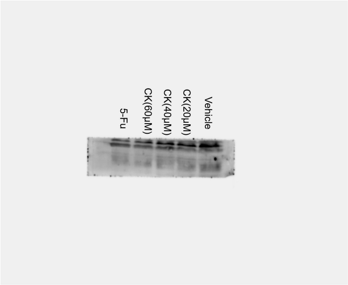

SLC7A11(2)

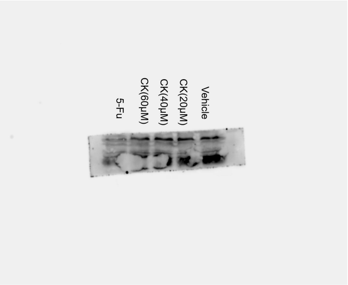

SLC7A11(3)

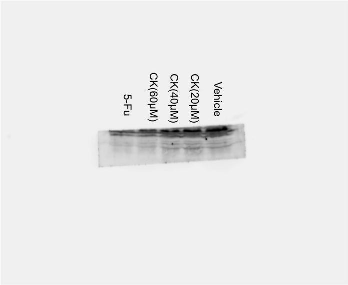

β-actin(1)

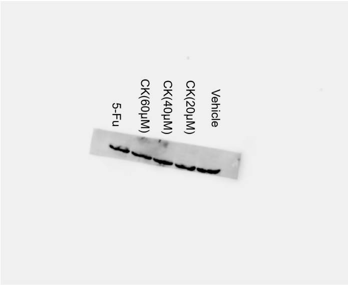

β-actin(2)

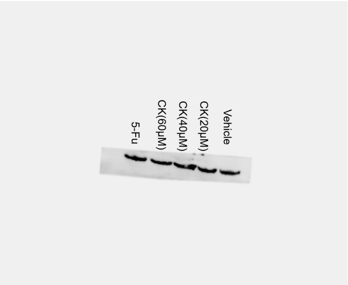

β-actin(3)

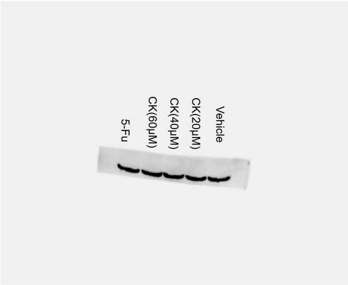

Fig.3(HepG2)

GPX4(1)

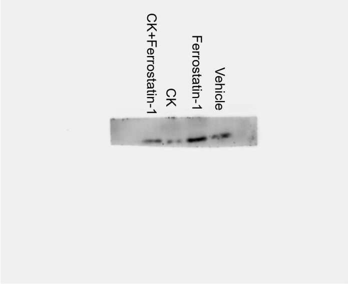

GPX4(2)

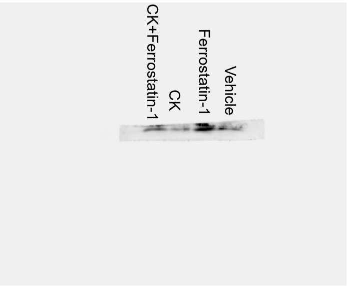

GPX4(3)

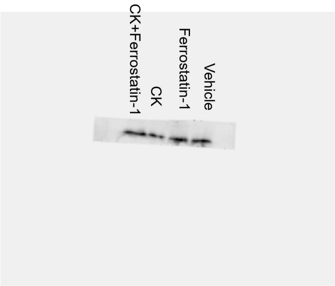

SLC7A11(1)

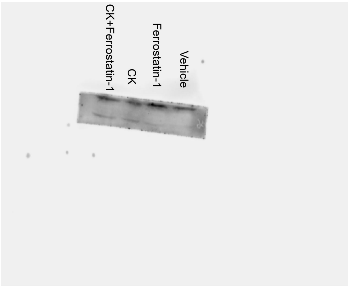

SLC7A11(2)

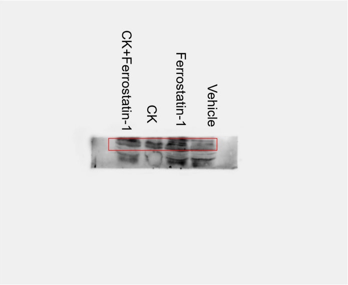

SLC7A11(3)

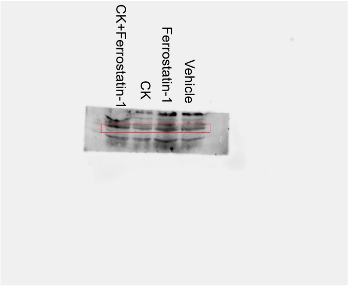

β-actin(1)

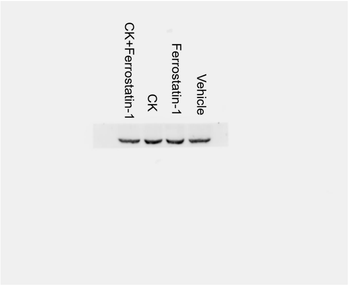

β-actin(2)

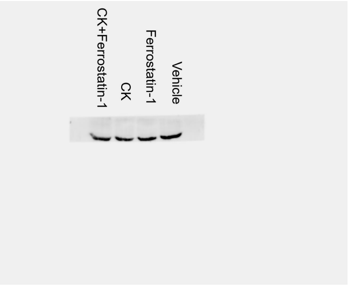

β-actin(3)

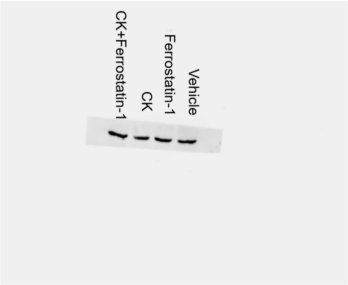

Fig.3(SK-Hep1)

GPX4(1)

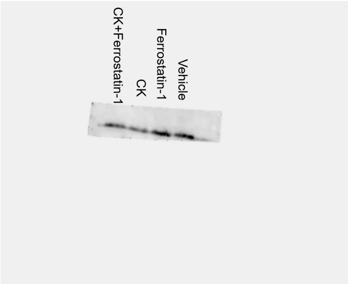

GPX4(2)

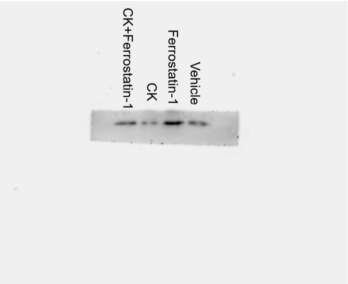

GPX4(3)

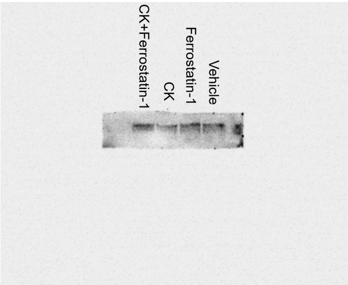

SLC7A11(1)

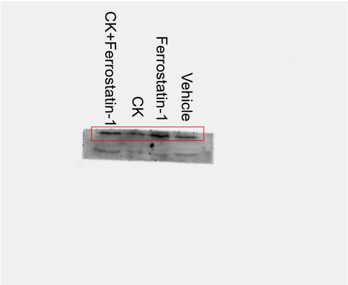

SLC7A11(2)

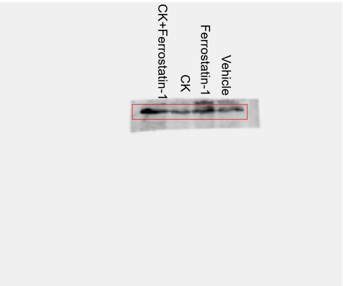

SLC7A11(3)

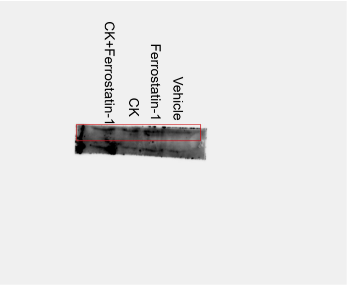

β-actin(1)

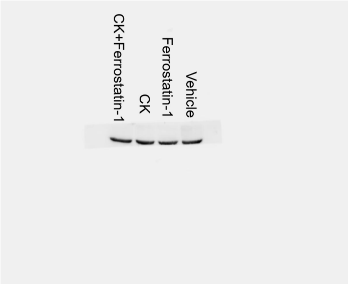

β-actin(2)

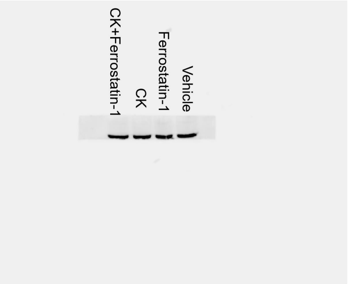

β-actin(3)

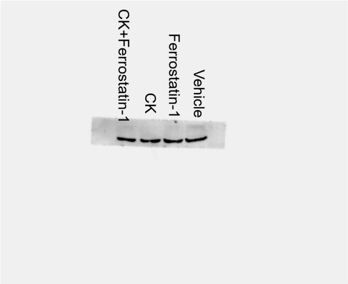

Fig.5A(HepG2)

FOXO1(1)

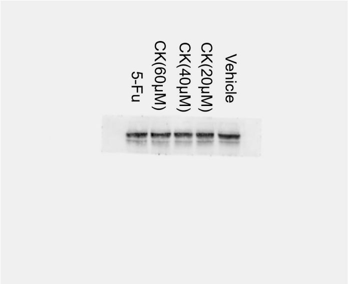

FOXO1(2)

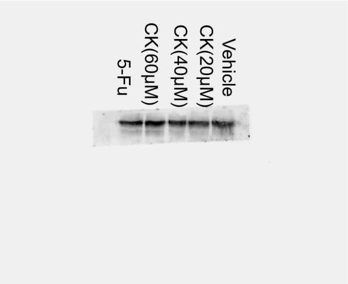

FOXO1(3)

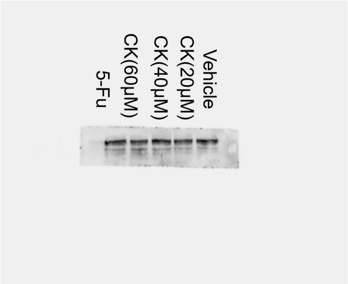

p-FOXO1(1)

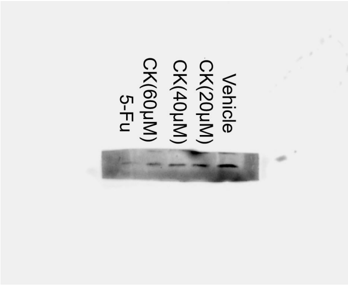

p-FOXO1(2)

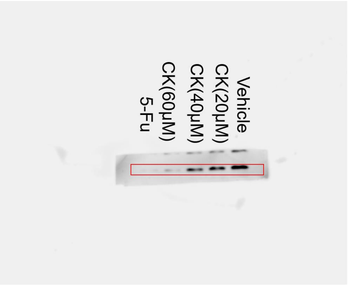

p-FOXO1(3)

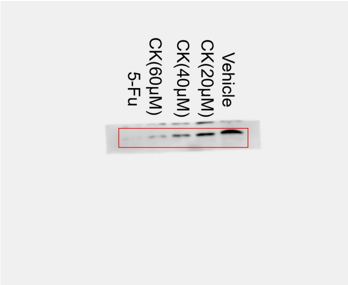

β-actin(1)

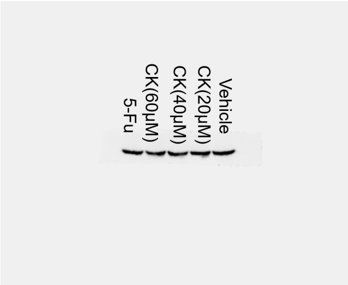

β-actin(2)

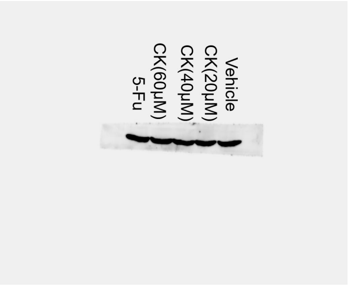

β-actin(3)

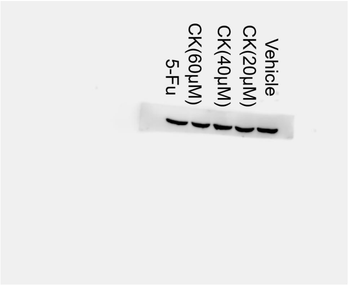

Fig.5A(SK-Hep1)

FOXO1(1)

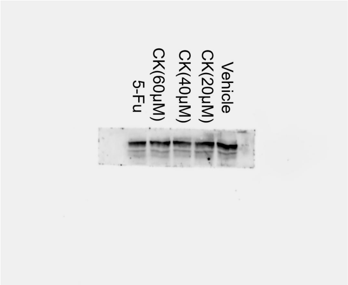

FOXO1(2)

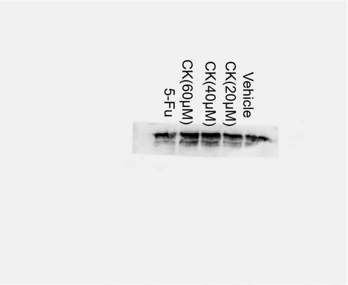

FOXO1(3)

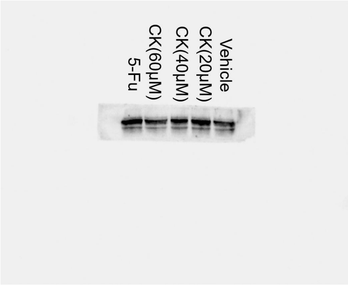

p-FOXO1(1)

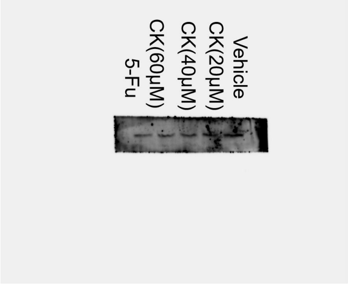

p-FOXO1(2)

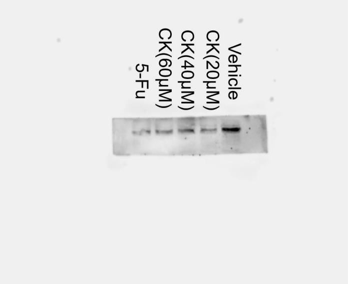

p-FOXO1(3)

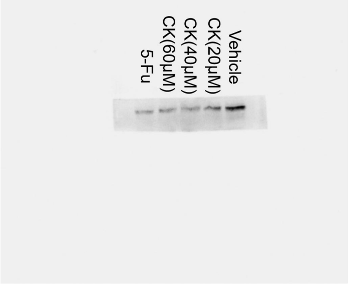

β-actin(1)

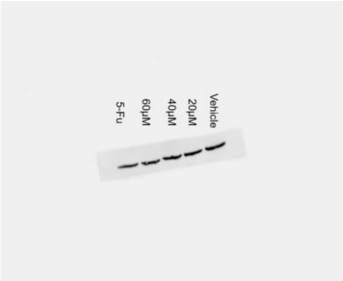

β-actin(2)

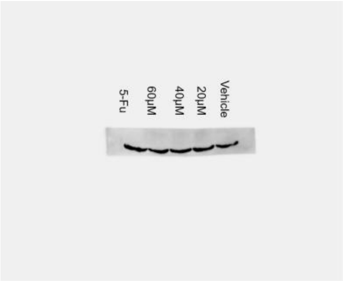

β-actin(3)

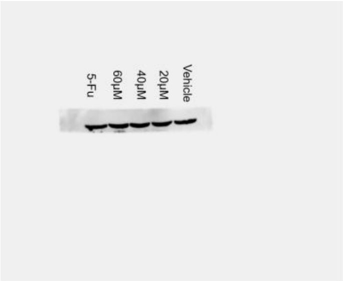

Fig.5B(HepG2)

GPX4(1)

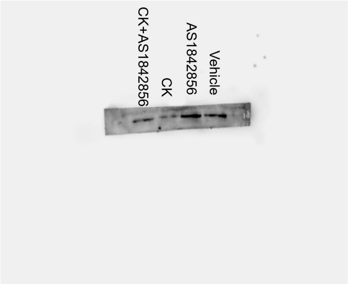

GPX4(2)

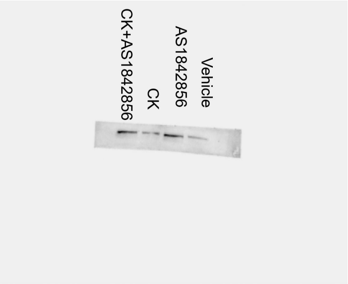

GPX4(3)

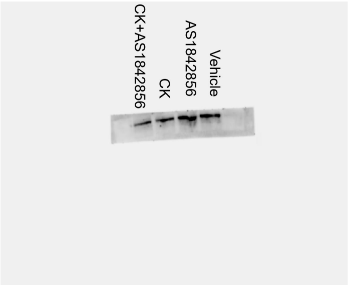

SLC7A11(1)

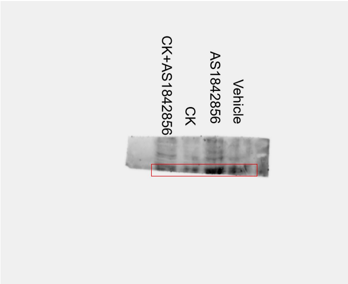

SLC7A11(2)

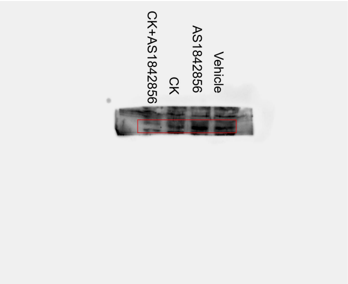

SLC7A11(3)

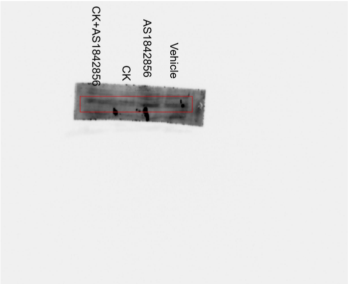

β-actin(1)

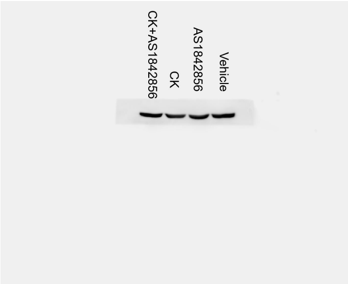

β-actin(2)

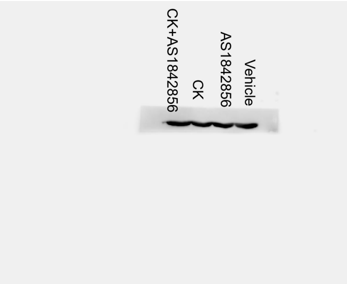

β-actin(3)

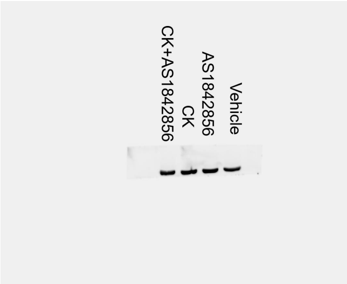

Fig.5B(SK-Hep-1)

GPX4(1)

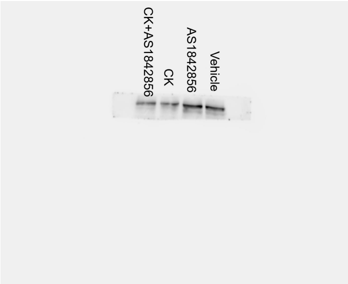

GPX4(2)

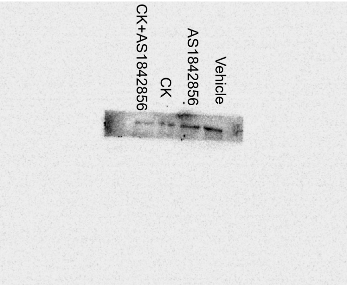

GPX4(3)

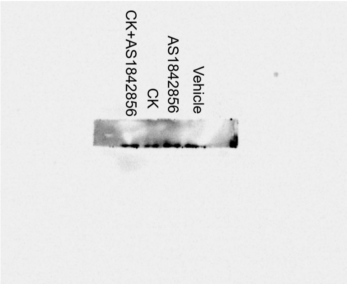

SLC7A11(1)

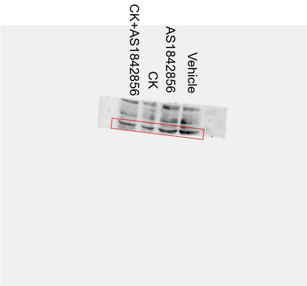

SLC7A11(2)

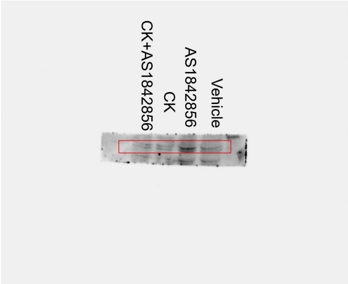

SLC7A11(3)

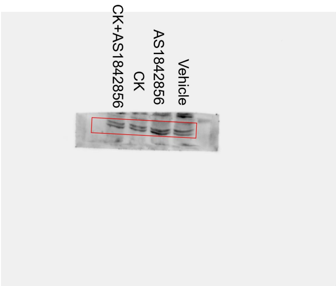

β-actin(1)

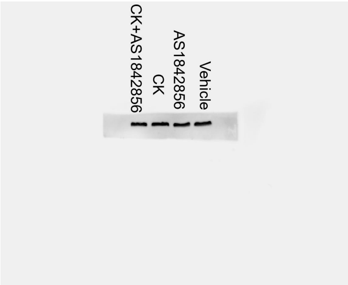

β-actin(2)

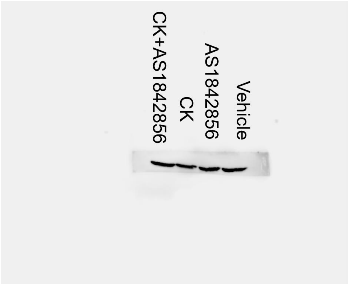

β-actin(3)

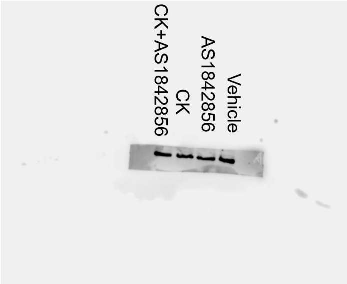

Fig.6

FOXO1(1)

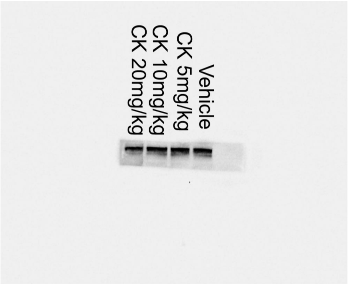

FOXO1(2)

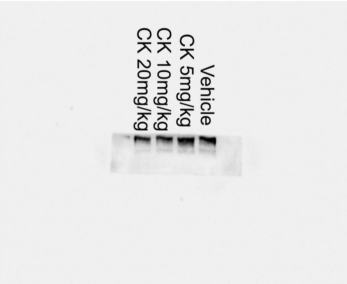

FOXO1(3)

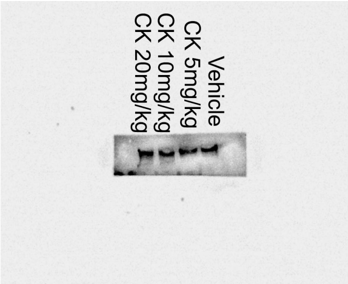

p-FOXO1(1)

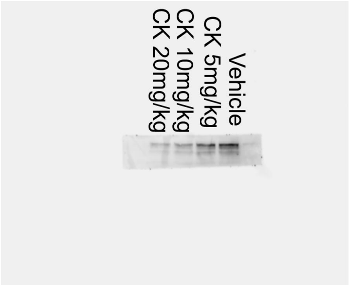

p-FOXO1(2)

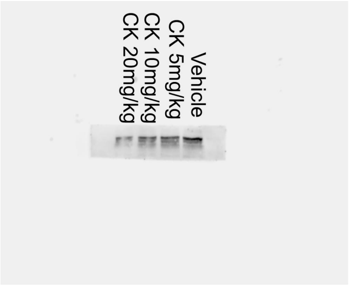

p-FOXO1(3)

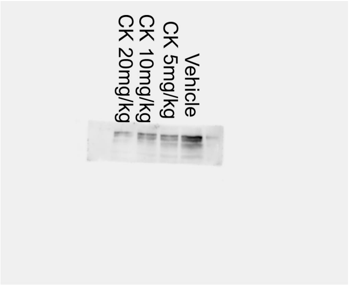

β-actin(1)

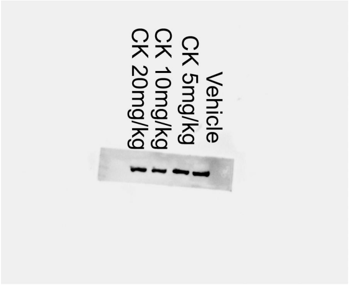

β-actin(2)

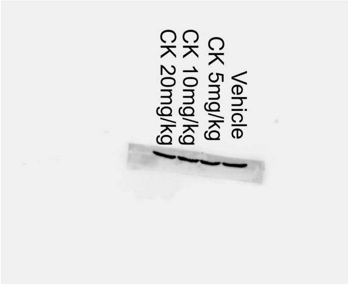

β-actin(3)

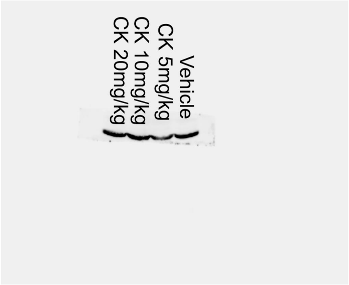

Supplement: Supplementary file 1 — Supplementary Material 1 [file 12906_2024_4471_MOESM1_ESM.pdf]
